# Identifying rapidly parasiticidal anti-malarial drugs using a simple and reliable in vitro parasite viability fast assay

**DOI:** 10.1186/s12936-015-0962-2

**Published:** 2015-11-05

**Authors:** María Linares, Sara Viera, Benigno Crespo, Virginia Franco, María G. Gómez-Lorenzo, María Belén Jiménez-Díaz, Íñigo Angulo-Barturen, Laura María Sanz, Francisco-Javier Gamo

**Affiliations:** R&D Alternative Discovery and Development, Diseases of the Developing World, GlaxoSmithKline, 28760 Tres Cantos, Madrid, Spain

**Keywords:** Drug discovery, Malaria, Parasiticidal, *Plasmodium falciparum*, Anti-malarial

## Abstract

**Background:**

The emergence of *Plasmodium falciparum* resistance to artemisinins threatens to undermine the effectiveness of artemisinin-based combination anti-malarial therapy. Developing suitable drugs to replace artemisinins requires the identification of new compounds that display rapid parasite killing kinetics. However, no current methods fully meet the requirements to screen large compound libraries for candidates with such properties. This study describes the development and validation of an in vitro parasite viability fast assay for identifying rapidly parasiticidal anti-malarial drugs.

**Methods:**

Parasite killing kinetics were determined by first culturing unlabelled erythrocytes with *P. falciparum* in the presence of anti-malarial drugs for 24 or 48 h. After removing the drug, samples were added to erythrocytes pre-labelled with intracellular dye to allow their subsequent identification. The ability of viable parasites to re-establish infection in labelled erythrocytes could then be detected by two-colour flow cytometry after tagging of parasite DNA. Thus, double-stained erythrocytes (with the pre-labelled intracellular dye and the parasite DNA dye) result only after establishment of new infections by surviving parasites. The capacity of the test anti-malarial drugs to eliminate viable parasites within 24 or 48 h could, therefore, be determined.

**Results:**

The parasite viability fast assay could be completed within 48 h following drug treatment and distinguished between rapidly parasiticidal anti-malarial drugs *versus* those acting more slowly. The assay was validated against ten standard anti-malarial agents with known properties and results correlated well with established methods. An abbreviated assay, suitable for adaption to medium–high throughput screening, was validated and applied against a set of 20 compounds retrieved from the publically available Medicines for Malaria Venture ‘Malaria Box’.

**Conclusion:**

The quantification of new infections to determine parasite viability offers important advantages over existing methods, and is amenable to medium–high throughput screening. In particular, the parasite viability fast assay allows discrimination of rapidly parasiticidal anti-malarial candidates.

## Background

*Plasmodium falciparum* malaria remains a formidable global health challenge. Although there have been recent improvements in mortality, 584,000 deaths were attributable to this parasite in 2013, and most of the victims were children [1].

At the end of the last century, *P. falciparum* resistance to the most clinically important anti-malarial agents had become widespread. The discovery and introduction of artemisinin-based combination therapy (ACT) salvaged the effectiveness of anti-malarial therapy. However, delayed parasite clearance in response to ACT has been observed clinically, and the development of parasite resistance to these agents should be anticipated [2]. The loss of artemisinin efficacy would threaten recent advances in malaria mortality and morbidity and compromise the malaria eradication agenda [3].

Artemisinins are rapidly parasiticidal, quickly relieving malarial symptoms and minimizing the within-patient window of opportunity for resistant parasite selection and subsequent dissemination [4]. Replacement of artemisinins requires the identification of new drugs with similar rapid parasite killing kinetics. Ideally, such properties need to be identified in the early stages of the drug development process. However, no current methods fully meet the requirements to screen large compound libraries for rapidly parasiticidal anti-malarial drug candidates.

Standard in vitro parasite growth inhibition assays expose parasites to an anti-malarial drug for a defined period of time and determine the remaining viable parasites by labelling nucleic acids or measuring enzymatic activity. However, these methods do not allow direct determination of the rapidity of parasite killing—metabolism is not always a good surrogate of parasite viability, and the reliability of such tests depends on the drug mechanism of action [5, 6]. Recently, a number of different approaches have been proposed for the assessment of parasite viability after drug treatment [7–10]. However, none of these methods provide a complete time course for parasite killing over a broad range of anti-malarial compounds with a variety of different modes of action.

In 2012, a low-throughput assay was developed at Tres Cantos, based on the direct measurement of *P. falciparum* parasite viability in response to drug treatment over time. This methodology quantifies accurately the number of parasites able to resume a productive growth after removal of the drug and addition of fresh erythrocytes. The method is based on limiting serial dilutions of treated parasites and their culture for up to 28 days to enable wells with a single viable parasite to render detectable parasitaemia. This standardized assay allows establishment of different parameters, such as the in vitro parasite reduction ratio (PRR) and the presence of a lag phase in response to treatment with a specific anti-malarial. Nevertheless, this protocol is very labour intensive and time consuming and difficult to adapt as a higher throughput assay [5].

This study describes the development and validation of an in vitro parasite viability fast assay for identifying rapidly parasiticidal anti-malarial drugs. The methodology has been adapted from a previously described invasion assay [11]. Parasite killing kinetics were determined by first culturing unlabelled erythrocytes with *P. falciparum* in the presence of anti-malarial drugs. Immediately following drug removal (at either 24 or 48 h), new erythrocytes were added which had been pre-labelled with intracellular dyes allowing their subsequent identification. Following incubation for 48 h, the ability of viable parasites to re-establish infection in labelled erythrocytes could then be detected by two-colour flow cytometry after labelling of parasite DNA, i.e., double-stained erythrocytes (with the pre-labelled intracellular dye and the parasite DNA dye) result only after establishment of new infections by surviving parasites. Thus, the capacity of the test anti-malarial drugs to eradicate viable parasites within 24 or 48 h could be determined.

Although different in vitro and in vivo assays have been established to measure re-invasion of *Plasmodium* parasites [11–13], this is the first time that such an assay has been used for determining the speed of parasite killing by anti-malarial compounds. This methodology provides a breakthrough over existing methods as it enables assessment of anti-malarial killing profiles 48 h following drug treatment and is amenable to automation. Thus, it should now be possible to interrogate large compound libraries to identify the next generation of anti-malarial drugs with artemisinin-like killing profiles [14–16].

## Methods

The final protocol is described below; optimization and validation of the assay is described in the “Results”.

### Chemicals and materials

Standard anti-malarial drugs were provided by GlaxoSmithKline and Medicines for Malaria Venture, except azithromycin which was purchased from USP (Rockville, MD, USA). Stock solutions were prepared in DMSO at 10 mM. The final DMSO concentration used in the experiments (<0.5 %) has no inhibitory effect on parasite cultures. Carboxylfluorescein diacetate succinimidyl ester (CFDA-SE) was purchased from Molecular Probes (California, USA), Hoechst 33342 and glutaraldehyde solution were purchased from Sigma (St Louis, MO, USA).

### In vitro culture of *Plasmodium falciparum* parasites

*Plasmodium falciparum* strain 3D7A was obtained from the Malaria Research and Reference Reagent Resource Center (MR4) and cultured using a modification of the method described by Trager et al. [17]. Briefly, parasites were cultured using RPMI-1640 supplemented with 0.5 % Albumax and 150 µM hypoxanthine at 2 % haematocrit under an atmosphere of 90 % N_2_, 5 % CO_2_, 5 % O_2_ at 37 °C. Erythrocytes were obtained from the Spanish Red Cross Blood Bank.

### Erythrocyte labelling

Erythrocytes were labelled with CFDA-SE. The protocol for erythrocyte labelling was optimized based on modification of a standard procedure [11]. Optimal conditions were incubation of the required volume of erythrocytes at 1 % haematocrit in RPMI 1640 with a CFDA-SE concentration of 10 µM at 37 °C for 2 h. The suspension was washed with complete medium and the labelled erythrocytes re-suspended to 1 % haematocrit with complete medium and incubated for 30 min at 37 °C. Finally, cells were washed twice with RPMI-1640. Labelled erythrocytes were diluted at 1 % haematocrit and stored at 4 °C for up to 24 h.

### Drug treatment and infection of labelled erythrocytes

**Fig. 1:**
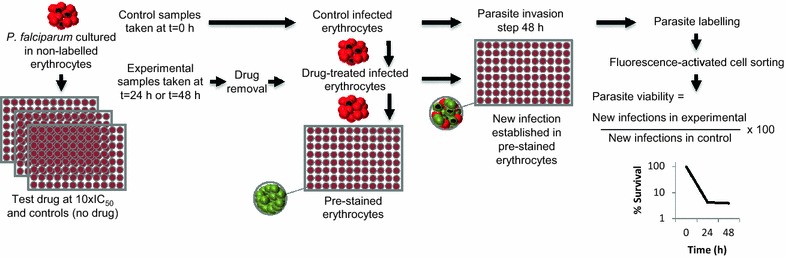
Scheme showing the in vitro parasite viability fast assay for identification of rapidly parasiticidal anti-malarial drugs. Parasite killing kinetics were determined by first culturing unlabelled erythrocytes with *P. falciparum* in the presence of anti-malarial drugs for 24 or 48 h. After removing the drug, samples were added to erythrocytes pre-labelled with intracellular dye to allow their subsequent identification. The ability of viable parasites to re-establish infection in labelled erythrocytes could then be detected by two-colour flow cytometry after tagging of parasite DNA. Thus, double-stained erythrocytes (with the pre-labelled intracellular dye and the parasite DNA dye) result only after establishment of new infections by surviving parasites. Controls samples containing no drug were progressed through the parasite invasion stage as per experimental samples to provide a value representing 100 % parasite viability at time 0

Assay methodology is summarized in Fig. 1. The conditions for the viability assay were chosen to mimic those used for standard IC_50_ determination (2 % haematocrit, 0.5 % parasitaemia with ≥80 % ring stages) [5]. Asynchronous cultures were incubated under shaking conditions to avoid multiple infections per erythrocyte. A culture volume of 50 μL per well with parasites at 4 % haematocrit and 0.5 % parasitaemia was dispensed into V-bottom, 96-well plates containing 50 μL of complete media with previously diluted drugs prepared at 2× their final concentration to give a final volume of 100 µL per well and a final drug concentration 10× the respective 50 % inhibitory concentration for *P. falciparum (Pf*IC_50_); this concentration has been shown in previous experiments to avoid sub-optimal drug exposure [5]. For the experimental plates, infected erythrocytes were exposed to drug for 24 or 48 h using standard incubation techniques at 37 °C. Drug was renewed every 24 h throughout the treatment period by retiring old media from the cultured wells and adding the same volume with fresh drug. Drug was removed following either 24 or 48 h of exposure. To remove drug, 80 µL of media containing drug was removed, leaving a 20-µL sample to which 200 µL of fresh media was added (1/10 dilution). Plates were centrifuged for 10 min at 600 g (Beckman Allegra X-12R), and 180 µL of media then removed and the remaining 20 µL, containing the infected erythrocytes, was re-suspended in 100 µL of complete media (1/5 dilution). Following drug removal, 70 µL of washed infected erythrocytes was immediately transferred to a new microtitre plate containing 130 µL of CFDA-SE-labelled, non-infected erythrocytes (1/3 dilution) at 2 % haematocrit in complete media. Plates were incubated for 48 h at 37 °C, 5 % CO_2_, 5 % O_2_, and 90 % N_2_ to allow new infections to develop in labelled erythrocytes as a surrogate measure of viable parasites. Control wells contained no drug and were exposed for 0 h before progression to the parasite invasion step using identical methods to those used for experimental plates.

### Parasite labelling

Following incubation, parasite cultures were washed with phosphate buffer saline (PBS). Plates containing 200 µL of cultured parasites were centrifuged and erythrocytes suspended with 2 µM Hoechst 33342 in RPMI 1640 and incubated at 37 °C for one hour in the dark. To allow the assay to be used for medium–high throughput screening, this method was modified by replacement of the medium with Hoechst solution (200 µL) and an incubation of 20 min. Cells were then fixed with 0.02 % glutaraldehyde and stored at 4 °C protected from the light until flow cytometry analysis.

### Fluorescence-activated cell sorting (FACS)

Following parasite staining, samples (60 µL), were transferred to cytometry tubes containing 300 µL of saline buffer (final haematocrit 0.3 %) and acquired in a LSRII flow cytometer [Becton–Dickinson (BD) Pharmingen, San Diego, CA, USA] equipped with a 355 nm 20 mW ultraviolet (UV) laser and a 488 nm 15 mW blue laser. Hoechst 33342 was excited by a UV laser and detected by a 450/40 filter. CFDA-SE was excited by a blue laser and detected by a 530/30 filter. Samples were analysed using FACSDiva software (Becton–Dickinson). In those samples with parasitaemia >0.1 %, the number of erythrocytes acquired was that which assured the gathering of at least 1000 events in the region established for infected erythrocytes. For parasitaemias <0.1 %, 8 × 10^5^ total erythrocytes were acquired.

### Data analysis and validation

Parasitized CFDA-SE-stained erythrocytes represent new infections that have been established from parasites surviving drug treatment. Thus, quantification of CFDA-SE-stained erythrocytes was used to evaluate parasite viability following drug treatment. In order to compare between different experiments, parasite viability was calculated as the percentage of infected CFDA-SE-stained erythrocytes in drug-treated samples at 24 or 48 h versus infected CFDA-SE-stained erythrocytes in concurrent control samples at time 0, i.e., controls allow normalization to a parasite viability of 100 % before drug treatment. The results were represented as the mean of this normalized percentage ± the standard error of the mean.

All statistical analyses were performed using the GraphPad Prism Software (version 6). The non-parametric test U-Mann–Whitney was used to compare the two groups under comparison. Differences between means were considered significant when the *P* value was <0.05.

## Results

### Optimization of erythrocyte staining

**Fig. 2:**
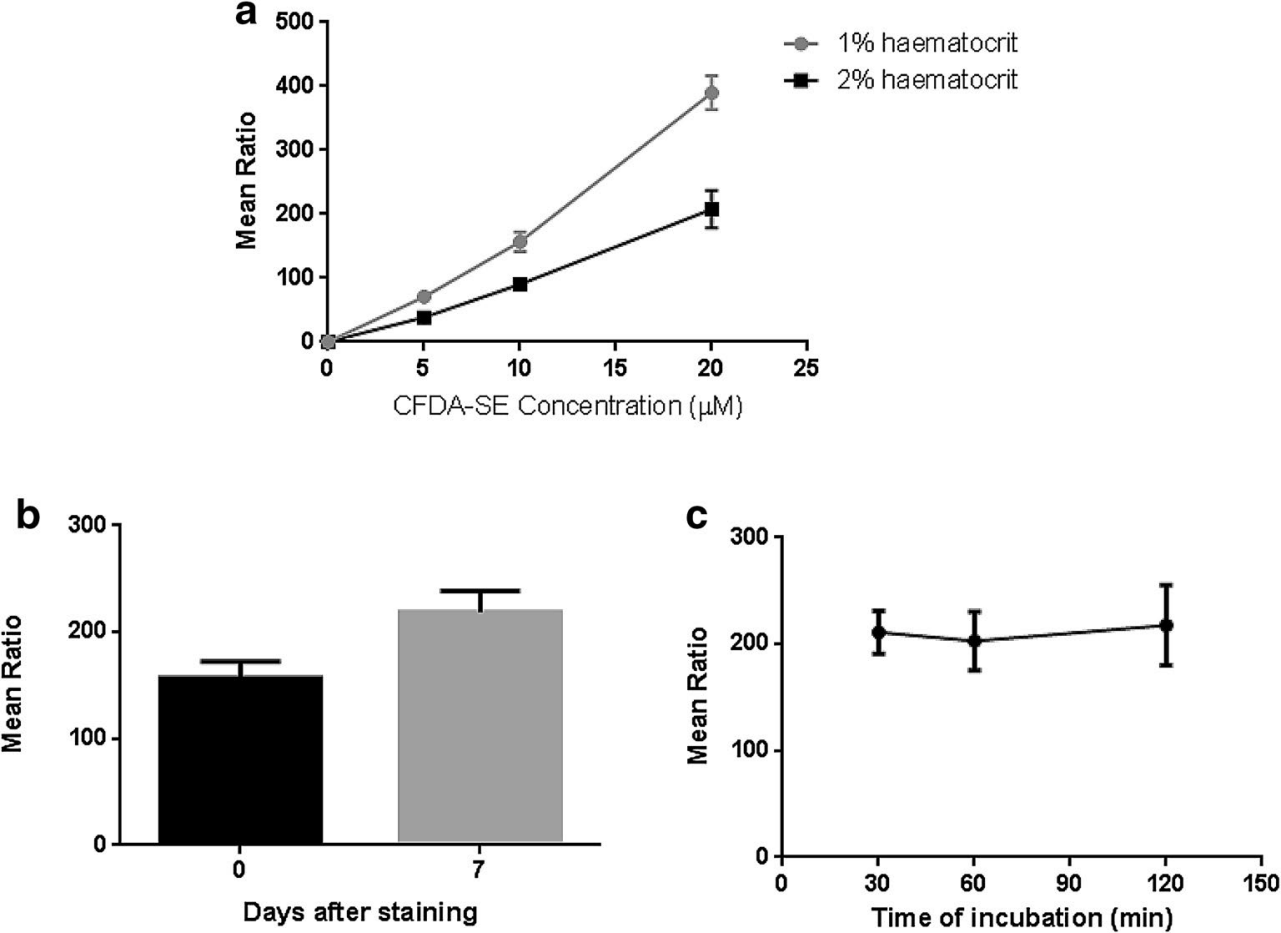
Optimization of erythrocyte labelling for fluorescence-activated cell sorting detection. *Graphs* represent the ratios of mean fluorescence intensities from positive cells with respect to the negative population. *Error bars* represent SEM for three replicates. **a** Optimization of the assay used different concentrations of CFDA-SE (20, 10 and 5 µM), were tested to stain erythrocytes using 1 or 2 % haematocrit. **b** Signal intensity by labelled erythrocytes remained robust for detection at least 7 days after staining (CFDA-SE 10 µM, 1 % haematocrit). **c** To test whether a reduction in incubation time could be achieved, different times (30 min, 1 and 2 h) were used for staining erythrocytes with 10 µM CFDA-SE at 1 % haematocrit

A previously described protocol for staining erythrocytes used erythrocytes labelled with 20 µM CFDA-SE for 120 min at 2 % haematocrit [11]. Optimization of this method tested decreasing concentrations of CFDA-SE (20 µM, 10 µM and 5 µM) at 1 or 2 % haematocrit. Concentrations of 10 µM CFDA-SE were sufficient for staining erythrocytes at 1 % haematocrit and enabled signal for detection (Fig. 2a) and labelling was stable for at least 7 days (Fig. 2b). Erythrocyte labelling incubation times were also tested (30, 60 and 120 min at 37 °C), with no significant differences in performance (Fig. 2c). Thus, a 30-min incubation time was selected.

### Estimating the rate of new infection by flow cytometry

To confirm that the optimized labelling conditions could detect new infections, *P. falciparum* cultures with 0.5 % parasitaemia were grown in the presence of labelled erythrocytes for 48 h. The final concentration of labelled erythrocytes was two-thirds of the total amount in the culture.

**Fig. 3:**
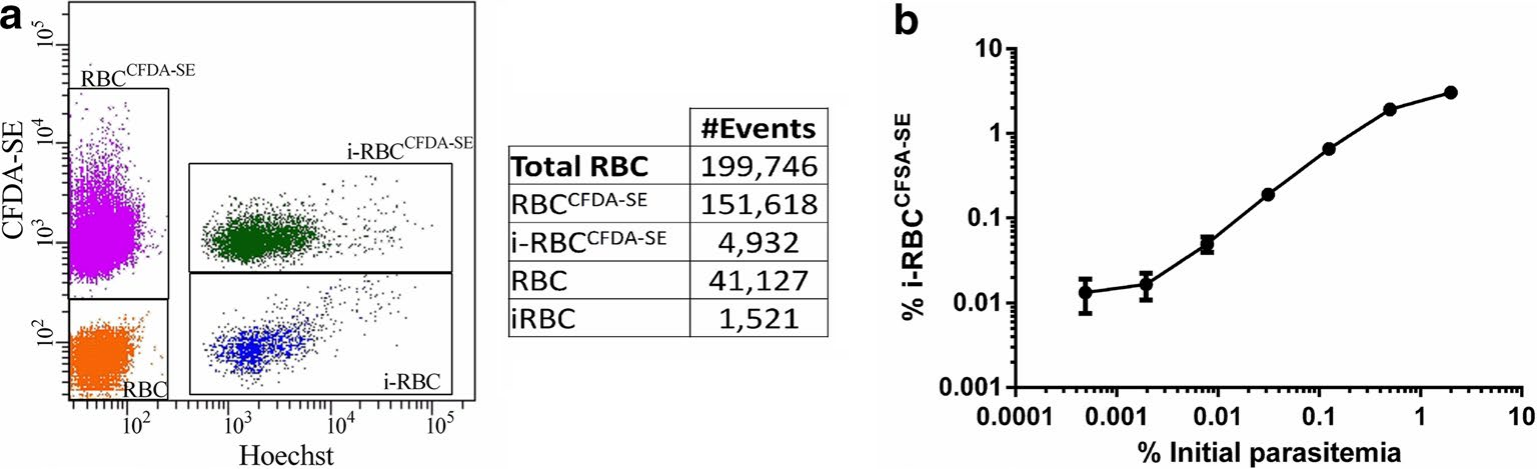
Fluorescence-activated cell sorting (FACS) analysis of infected erythrocytes labelled with CFDA-SE. **a** DNA staining with Hoechst of parasites grown in the presence of CFDA-SE labelled erythrocytes allows robust detection of four distinct cellular populations. Data are representative of two independent experiments. **b** A *P. falciparum* 3D7 inoculum grown in standard culture conditions at 2 % haematocrit and 2 % parasitaemia was diluted (fourfold serial dilutions), in fresh media containing labelled non-infected erythrocytes for 48 h. Parasites were labelled with Hoechst and fluorescence of i-RBC^CFDA-SE^ quantified by FACS. *Y axis* represents % of i-RBC^CFDA-SE^ with respect to total number of RBC. *Error bars* represent SEM for three replicates. Established conditions allow detection of initial parasitaemia as low as 0.002 %

Flow cytometry analysis revealed four different erythrocyte populations (Fig. 3a: non-infected non-stained erythrocytes (RBC), infected non-stained erythrocytes (i-RBC), non-infected stained erythrocytes (RBC^CFDA-SE^) and infected stained erythrocytes (i-RBC^CFDA-SE^). Although i-RBC could result from initial or new infections of non-stained erythrocytes, all instances of i-RBC^CFDA-SE^ must result from new infections. Thus, the quantification of i-RBC^CFDA-SE^ can be used to determine parasite viability following drug treatment.

As previously described, there was no difference in the rate of infection between pre-stained and non-stained erythrocytes [11]. In fact, as can be seen in Fig. 3a, the ratio between i-RBC^CFDA-SE^ and i-RBC did not differ significantly from the ratio between RBC^CFDA-SE^ and RBC, indicative that both populations of erythrocytes (RBC^CFDA-SE^ and RBC) were equally susceptible to invasion. This is important because, as samples of treated, non-labelled erythrocytes are diluted three-fold by adding labelled erythrocytes, as much as two-thirds of the new infections can be quantified, but not the approximately one-third of infections that are produced in the initial, non-stained erythrocytes. As no bias was detected, the total number of new infections can be estimated from the quantified events, i.e., total infections = 3/2 i-RBC^CFDA-SE^.

### Dynamic range of the assay

The dynamic range of the technique was estimated by growing serial dilutions of an initial *P. falciparum* 3D7A culture at 2 % parasitaemia (2 % haematocrit) in the presence of RBC^CFDA-SE^ for 48 h. As it is shown in Fig. 3b, established conditions allowed detection of initial parasitaemia levels as low as 0.002 %. Fluorescent quantification of i-RBC^CFDA-SE^ was proportional to the number of initial parasites in the range of 0.5–0.005 % parasitaemia. This dynamic range allows clear differentiation between fast-acting compounds and moderate/slow anti-malarial agents.

### Assay validation

**Fig. 4:**
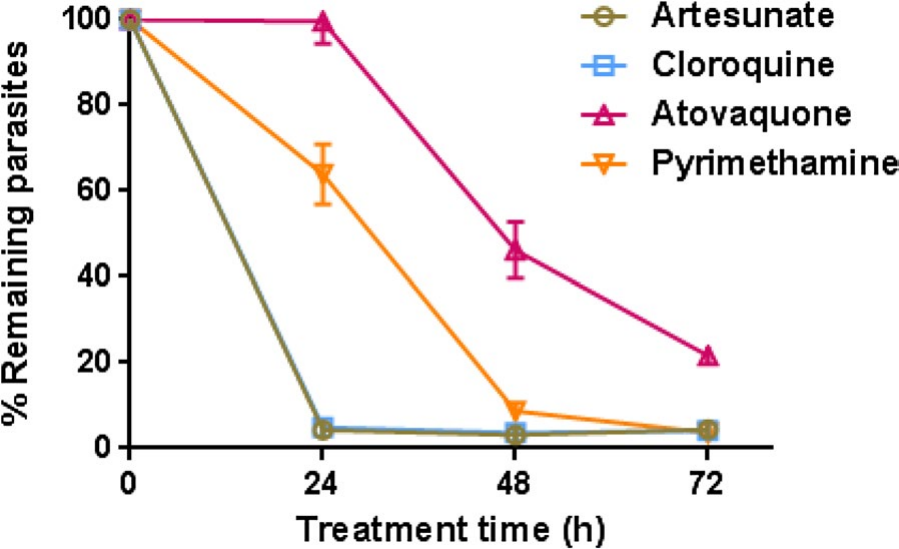
Determination of killing rate profile for different classical anti-malarial drugs. *Plasmodium falciparum* viability time-course profile for artesunate, chloroquine, atovaquone, and pyrimethamine at 10 × IC_50_. Fast-acting anti-malarial drugs (artesunate and chloroquine) can be readily distinguished from moderate or slow-acting compounds (pyrimethamine and atovaquone). The number of viable infected erythrocytes was normalized using the number of untreated parasites. *Error bars* represent SEM for 4–9 replicates

The assay was validated against four anti-malarial agents with well-characterized parasite killing profiles: atovaquone and pyrimethamine with a slow/moderate rate of killing and chloroquine and artesunate with rapid parasite killing. As shown in Fig. 4, the assay was able to distinguish between fast- and slow-acting agents within 48 h after drug treatment. Consequently, 24 and 48 h were identified as the most relevant time points that could provide the information required to unequivocally identify rapidly parasiticidal compounds. These results correlate well with killing rate profiles reported using PRR methodology and equally can be attributed to the different anti-malarial modes of action tested [5].

### Adaptation for medium–high throughput screening

This new methodology is more amenable for medium–high throughput than the standard PRR assay [5]. Killing profiles of different drugs can be determined in only 48 h after drug treatment instead of the 3–4 weeks required to detect single viable parasite using the limiting dilution method [5].

The following modifications were tested: (1) parasites were directly stained without removing the culture media, and, (2) culture media was replaced by Hoechst solution. Additionally, different incubation times (10, 15 and 20 min) were tested before fixation. An incubation time of 20 min when the medium was replaced by Hoechst solution was selected as optimal, and used for subsequent experiments. Evaluation of atovaquone, pyrimethamine, chloroquine, and artemisinin using the abbreviated format produced similar results as for the initial format.

**Fig. 5:**
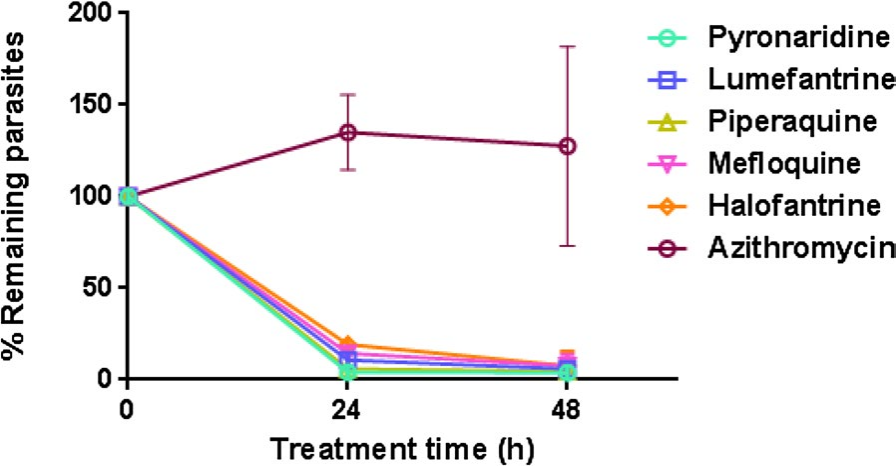
Two-time point viability analysis using an abbreviated procedure. *Plasmodium falciparum* cultures were treated with indicated drugs and parasites sampled after 24 and 48 h. Medium-throughput conditions allowed discrimination of fast-acting profiles of pyronaridine, halofantrine, lumefantrine, piperaquine and mefloquine from the azithromycin anti-malarial profile, with parasite killing only in the second generation. The number of viable infected erythrocytes was normalized using the number of untreated parasites. *Error bars* represent SEM for 4–9 replicates

To further validate the abbreviated format, a panel of six additional standard anti-malarial molecules was also tested (Fig. 5). As expected, mefloquine, piperaquine, lumefantrine, halofantrine, and pyronaridine showed a fast-acting profile. However, azithromycin did not show any effect on parasite viability, consistent with its mode of action: azithromycin affects protein synthesis in the *Plasmodium* apicoplast and anti-malarial effects are only observed after two cycles of intra-erythrocytic development [18].

Furthermore, the abbreviated assay was further validated and applied to identify novel rapidly parasiticidal anti-malarial agents. A set of 20 compounds retrieved from the Medicines for Malaria Venture ‘Malaria box’ [19], was tested with the parasite viability fast assay and the previously described in vitro killing rate assay using limiting dilutions [5]. The profiles of rapidly parasiticidal anti-malarial agents (chloroquine/artemisinin-like) were clearly differentiated from those of slow (atovaquone-like) or moderate (pyrimethamine-like) compounds and results were confirmed independently of the method used (Table 1). None of the compounds that displayed a fast parasite killing profile was previously known to have this property. Thus, these potentially represent new avenues of investigation for the development of novel anti-malarial agents which have parasite killing kinetics similar to artemisinin.

**Table 1:** Comparison between the parasite viability kinetic assay and the in vitro killing rate assay based on limiting dilutions using compounds from the Medicines for Malaria Venture Malaria Box

Compound	In vitro killing rate assay (standard assay)	Viability assay (fast assay)
MMV000478	Fast	Fast
MMV665939	Slow	Slow
MMV019066	Slow	Slow
MMV019662	Slow	Slow
MMV007564	Moderate	Fast/moderate
MMV020750	Moderate	Moderate
MMV665789	Moderate	Moderate
MMV665928	Fast	Fast
MMV006767	Moderate	Moderate
MMV008149	Slow	Slow
MMV009108	Slow	Slow
MMV665924	Fast	Fast
MMV011895	Fast	Fast
MMV019017	Fast	Fast
MMV009063	Fast	Fast
MMV665794	Moderate	Moderate
MMV665824	Slow	Slow
MMV006455	Fast	Fast
MMV665852	Moderate/fast	Fast
MMV665888	Moderate	Moderate

*Fast* chloroquine/artemisinin-like (no viable parasites detected after 24 h’ treatment), *moderate* pyrimethamine-like (no viable parasites detected after 48 h’ treatment), *slow* atovaquone-like (more than 48 h’ treatment needed to kill all parasites)

## Discussion

Artemisinin resistance is emerging and threatens to undermine the effectiveness of anti-malarial therapy. Consequently, identifying anti-malarial drugs that could succeed artemisinins, and which retain their key property of rapid killing of *Plasmodium* is a priority.

The parasite viability fast assay described in this paper is an important step to enable the identification of rapidly parasiticidal anti-malarial agents. Recent efforts have identified numerous leads for new anti-malarial agents [14–16]. However, their evaluation for artemisinin-like parasite killing kinetics has been limited, as current methods are time consuming and cannot easily be scaled to screen the thousands of candidate molecules.

The parasite viability fast assay described here was able to distinguish between anti-malarial agents with artemisinin-like parasite killing kinetics and those that act more slowly within 48 h following drug treatment. The assay was validated against standard anti-malarial drugs with known properties and results correlated well with established methods.

The availability of a medium–high throughput assay for identifying compounds with artemisinin-like parasite killing kinetics is a critical step in developing the next generation of anti-malarial therapy. Thus, the assay described here has been adapted to allow its application in medium–high throughput screening and has been evaluated against a test set of candidate compounds. The methodology lends itself to automation, using simple incubations and washing steps without the labour-intensive procedures used for other assays, such as the limiting dilution method. Staining stability allows storage, and multiple samples can be simultaneously assayed. It is envisaged that the assay could be used for screening compound libraries such as the Tres Cantos Antimalarial Set (TCAMS), which includes nearly 13,500 compounds. The parasite viability kinetic assay could be used with any laboratory parasite strain, including adapted ex vivo isolates. This could be particularly relevant in the evaluation of artesunate derivatives, as resistant parasites do not present any change in terms of in vitro IC_50_, and complex read-outs are required [20, 21].

The parasite viability fast assay described here has a less sensitive limit of detection (equivalent to 0.002 % parasitaemia) compared with the limiting dilution method (equivalent to 0.000005 % parasitaemia) [5]. Thus, any compound that elicited a decrease in parasitaemia of >99 % would have the same profile in the assay. Although this limit of detection is sufficient to differentiate between fast-acting compounds and moderate/slow-acting ones, it cannot resolve differences between two fast-acting compounds (e.g., artemisinin and chloroquine). A further limitation of the methodology is that it cannot evaluate the parasiticidal effects of compounds that display a delayed death phenotype, for example, azithromycin. However, molecules with such a mechanism of action can be identified using standard evaluations at 48 and 96 h to prove a shift in the IC_50_. Alternatively, the methodology described here could be adapted by extending the incubation time with pre-labelled erythrocytes to cover two parasite lifecycles.

The parasite viability fast assay offers a rapid, direct measurement of parasite killing that does not rely on parasite metabolic activity or accumulation of specific molecules. Thus, potential bias or artefacts that appear when viability and parasite metabolic activity become uncoupled are avoided. Although other assays have been developed for determining parasite killing kinetics, they use indirect measurements to assess parasite viability [8, 9]. For example, the rapid assay described by Le Manach uses a modification of the standard [^3^H] hypoxanthine incorporation assay for IC_50_ calculations [9]. However, atovaquone and pyrimethamine do not display the expected profile corresponding to their mechanism of action [9]. A method that detects shifts in parasite mRNA levels has been described by Bahamontes-Rosa [8], but can only differentiate between cidal or static behaviours and cannot evaluate the speed of parasite killing. The method is also laborious and time consuming, reporting results at 240 h post-treatment [8].

Exceptional efforts have greatly expanded the pool of potential candidate molecules with anti-plasmodial activity [14–16]. The main challenge now is to rapidly process those candidates to identify the next generation of anti-malarial therapy. The parasite viability fast assay described here is robust, reliable, rapid, expandable, and could be easily automated. The methodology requires no specialized equipment and can be easily implemented in standard laboratories. The assay can also be used with different parasite strains, offering a flexible research tool. The quantification of new infections to assess parasite viability offers important advantages over existing methods, and in particular allows discrimination of rapidly parasiticidal anti-malarial candidates that have the potential to succeed the artemisinins.
